# Stain removing, juice-clarifying, and starch-liquefying potentials of amylase from *Pleurotus tuberregium* in submerged fermentation system

**DOI:** 10.1186/s43141-022-00298-4

**Published:** 2022-02-10

**Authors:** Comfort Olukemi Bamigboye, Raphael E. Okonji, Iyanu Oluwalonimi Oluremi, Victoria James

**Affiliations:** 1 Microbiology Unit, Department of Pure and Applied Biology, Ladoke Akintola University of Technology, Ogbomoso, P.M.B. 4000 Nigeria; 2 Department of Biochemistry and Molecular Biology, Obafemi Awolowo University, Ile-Ife, Nigeria

**Keywords:** Amylase, *Pleurotus tuberregium*, Stain removal, Juice clarification, Submerged fermentation, Amylase characterization, Mushroom

## Abstract

**Background:**

Amylase is used commercially in food, textiles, sugar syrup, paper, and detergent industries. Bacteria and fungi remain a significant source of industrial enzymes. *Pleurotus tuberregium* is a macro-fungi that can exist as a fruiting body, sclerotium, mycelium, and spores. Some studies have been conducted on this fungus, with minimal studies on its enzyme activity (s) using the submerged fermentation technique.

**Results:**

The purified amylase has a specific activity of 5.26 U/mg, total activity of 189.20 U, maximally active at 70 °C, pH of 5, and retaining 100% of its activity at 30 ^o^C for 4 min. *P. tuberregium* amylase showed optimal activity with plantain peel, followed by starch and pineapple peel (42, 30, and 29 μg/mL/min respectively). The presence of Ca^2+^, Mg^2+^, and Na^+^ ions in the reaction mixture activated the enzyme activity*,* but was slightly and moderately inhibited by KCl and Na_2_H_2_PO_4_ respectively. The crude enzyme effectively clarified juice, liquefied soluble cassava starch (with a release of appreciable glucose quantity), and partially de-stained white fabric.

**Conclusions:**

The amylase obtained from the submerged fermentation of *Pleurotus tuberregium* has potential applications in food and detergent industries.

## Background

Amylases are mainly hydrolases hydrolyzing glycosidic bonds in starch molecules. Broadly, two types are mainly recognized, exo- and endo-amylases. The former hydrolyses the non-reducing end of starch while the latter hydrolyses glycosidic linkages within the starch molecule [1]. There are three main subtypes of amylases, α, β, and γ–amylases. γ–amylases have a pH optimal of 3, β-amylases functions around 4.0–5.5, while α-amylases acts at pH 7 with maximum activity at 6.0 [2]. α-amylases have high substrate specificity and are calcium-dependent enzymes.

Industrially, amylases account for about 30% of the world’s enzyme market. About 90% of liquid detergents for dishwashing and laundry contain amylases where it removes tough stains and degrades starchy food residues to dextrins and smaller oligosaccharides [3]. α-amylases are used in starch industry for liquefaction, where the partial hydrolysis of starch residues leads to the production of short chain dextrin. This lowers the viscosity of the starch solution and is further applied in the paper industry for hydrolyzing high molecular weight starch in coated paper. This improves the smoothness and strength of the paper. Further, α-amylases have found application in fuel alcohol production and textile industry.

Microbial amylases are cost-effective, and therefore in high demand [4, 5]. They can meet industrial demand with the possibility of increasing the levels of the microbial enzyme through genetic engineering techniques, induction, continuous culture selection, and optimization of growth conditions [6]. Microbial amylases have some specific properties which make them qualified for use in various industries. These properties include differences in optimum pH, thermotolerance, and optimum temperature [7]. Fungal enzymes are secreted extracellularly, with improved ease of isolation making fungi a more economical source of amylase compared to others [8]. However, this depends mainly on the microbial strain, nutrient requirement, cultivation methods, metal ions, cell growth, thermostability, incubation time, pH, and temperature.

Submerged fermentation is the preferred technique for producing commercial enzymes of microbial origin. Some important fungal metabolites have been produced successfully using this technique [9]. An advantage of submerged fermentation over solid-state fermentation is that the process parameters (temperature, pH, and dissolved oxygen) can be easily monitored and controlled. Additionally, scale-up of the process is easy and simple microscopic analysis can be used to monitor contamination and enzymes are easily extracted [2]. Heterogeneity and substrate insolubility makes this problematic in solid-state fermentation [10, 11].

Laccase was recently purified and characterized from an edible fungus-*P. tuberregium*, with special dye degrading activities [12]. To the best of our knowledge, there is limited or no report on the potential industrial applications of α-amylase from *P. tuberregium*. α-amylase reported previously from fungi are mainly from genus *Aspergillus*, *Trichoderma*, *Thermomyces*, and *Penicillium* [2]. Therefore, this work aimed to partially purify and characterize α-amylase from *P. tuberregium* in submerged fermentation system and establish some of its potential applications in detergent, starch and food industries.

## Methods

### Organism and maintenance

*Pleurotus tuber-regium* culture was obtained from the Microbiology Research Unit of Pure and Applied Biology and maintained throughout the experiment on the potato dextrose agar slant. This *P. tuber-regium* strain was earlier characterized and identified using molecular techniques [13].

### Chemicals and reagents

Citric acid, sodium citrate, ammonium sulfate (enzyme grade), potassium chloride, and ɛ-amino-n-caproic acid were from BDH Chemical Limited, Poole, England. Ethanol, 3,5-dinitrosalicylic acid, and soluble starch were Sigma-Aldrich Co., St Louis, USA, Coomassie Brilliant-Blue, and Bovine Serum Albumin from Sigma Chemical Company, St Louis, Mo., USA. CM-Sephadex C-25 was from Pharmacia Fine chemical, Uppsala, Sweden. Disodium Hydrogen phosphate and monosodium dihydrogen phosphate were products of Kermel Reagent Company Limited, Tianjin, China. Other chemicals, solvents, and media ingredients used for the experimental purpose were of analytical grade and were procured from reputed chemical companies.

### Media preparation and submerged fermentation

Culture media was prepared by modifying the method of Bamigboye et al., optimized for the cultivation of *P. tuber-regium* [14]. Precisely 0.38 g/l yeast extract agar, 0.1 g/l of potassium dihydrogen phosphate (KH_2_PO_4_), and 0.06 g/l of magnesium sulfate (MgSO_4_.7H_2_O) were weighed into distilled water in a 250 mL conical flask. Precisely 1.8 g/l of soluble cassava starch was added to the culture medium as the primary carbon source for amylase. The prepared solution was autoclaved for 15 min at 121 °C. Five agar plugs of a seven-day-old culture of *P. tuber-regium* were used to inoculate the prepared media, incubated at 35 °C for 21 days.

### Enzyme extraction and characterization

All the analyses were performed at temperatures between 0 and 35°C. The mycelia were separated from the submerged culture using cheesecloth, with the filtrate serving as the crude amylase. After that, the filtrate was studied for protein concentration and amylase activity.

#### Alpha-amylase assay

The amylase activity was measured using the method of Miller with minor modifications [15]. The reaction mixture contained 0.1 mL of 1% viscous starch and 0.05 mL of crude enzyme extract. The mixture was incubated at 37 °C for 30 min. The control tubes (enzyme blank) contained an equal quantity of the substrate and 0.05 mL of distilled water. The control and experimental tubes were incubated at the same temperature and time. The reaction was stopped by dispensing 1.0 mL of 3,5-dinitrosalicylic acid reagent into the mixture. The mixture’s temperature was raised to 100 °C for 5 min, cooled, and optical density measurement was taken at 540 nm. A unit of alpha-amylase activity was described as the quantity of enzyme required to liberate reducing sugar equivalent to 1 μmol glucose per minute under the indicated analysis conditions. A standard calibration curve of glucose was plotted and used for the estimation [16].

#### Determination of protein concentration

Bovine serum albumin was used as the standard to estimate protein concentration, with the protein absorbance interpolated from a standard protein curve. The reaction mixture consisted of a Bradford reagent (1.0 mL) and the enzyme solution (10 μl); the absorbance was read at 595 nm [17].

#### Ammonium sulfate precipitation

The crude enzyme was brought to 80% ammonium sulfate saturation (560 g/L) by adding and stirring solid ammonium sulfate. This was done for 1 h with occasional stirring until all the salts were completely dissolved in the supernatant. The mixture was maintained at 4 °C for 12 h, centrifuged for 30 min at 4000 rpm. The supernatant was thrown out, and the precipitate was collected and re-suspended in a small amount of 0.2 M phosphate buffer (pH 7.5) [18].

#### Desalting by dialysis

The ammonium sulfate precipitate was dialyzed against several changes of 0.2 M phosphate buffer, pH 7.5 for 18 h. The dialysate was centrifuged at 4000 rpm for 30 min to remove insoluble components, and the supernatant was assayed for alpha-amylase activity and protein concentration.

#### Sephadex C-25 ion exchange chromatography

Sephadex C-25 cation exchanger was pretreated by soaking 20 g of resin in 1 L of distilled water for 24 h. The resin was washed with 15 volumes of 0.5 M NaOH for 30 min. A series of stirring and decantation followed this until effluent had attained a pH of 8.0. It was later washed with 15 volumes of 0.5 M HCl followed by continuous washing with distilled water until the effluent was at pH 7 and later liberated with 0.2 M phosphate buffer with pH 7.5. A column (1.5 × 10 cm) of Sephadex C-25 was packed and equilibrated with 0.2 M phosphate buffer, pH 7.5. The column was layered with the dialyzed protein from the preceding step and washed with 0.2 M phosphate buffer, pH 7.5 to remove unbound proteins, followed by elution with an 80 mL linear gradient of 0–1 M NaCl in 0.2 M phosphate buffer, pH 7.5. Fractions of 2.0 mL were collected from the column with a flow rate of 30 mL per hour. Protein was monitored by the Bradford method. The fractions were also assayed for alpha-amylase activity. The active fractions from the column were pooled and dialyzed against 50% glycerol in a 0.2 M solution of phosphate buffer (pH 7.5). The dialyzed fraction was assayed for alpha-amylase activity and protein concentration. The enzyme obtained was used for other studies.

### Effect of pH on the enzyme activity

The effect of pH on the enzyme activity was determined by analyzing the enzyme activity using different buffers: 50 mM citrate buffer (pH 3–5); 50 mM phosphate buffer (pH 6.0–7.0), 50 mM Tris buffer (pH 8.0–9.0) and 50 mM borate buffer (pH 10.0). The alpha-amylase activity was assayed by preparing 1% starch in the different buffer solutions.

### Optimum temperature

Amylase was assayed at temperatures between 30 °C and 100 °C to determine the effect of temperature on enzyme activity and establish the optimum temperature of the enzyme. The assay mixture was first incubated at the indicated temperature with an aliquot of the enzyme, equilibrated at the same temperature. The amylase activity was assayed routinely, as previously described.

### Heat stability

The heat stability of the enzyme was determined between 30 °C and 60 °C. The enzyme was incubated at a particular temperature, and an aliquot was taken for enzyme assay at 15 min intervals for 1 h.

### Effect of metal ions on the enzyme activity

The effect of metal ions on *P. tuber-regium* amylase was tested. The ions tested were Na^+^, K^+^, Ca^2+^, Mg^2+^, and Fe^3+^ at 0.1 mM, 0.5 mM, and 1.0 mM in a typical amylase assay mixture. The metal salts were dissolved in distilled water, and the reaction mixture without the salts was taken as control with 100% activity.

### Substrate specificity

The enzyme’s substrate specificity was determined using different compounds, including pectin, starch, pineapple peel, and plantain peel at 1.0 % concentrations in a typical alpha-amylase assay mixture.

### Potential industrial applications of crude amylase from *P. tuber-regium*

#### Removal of sand and ink stain from fabric

This was done by a modified method of Saini et al.; two clean white fabrics (8 cm–40 cm) were stained with wet sand and ink (equal amount), soaked in 15 mL of crude amylase, or water for the control, after which the fabric was washed and visually observed for 10 min [19].

#### Juice clarification

Fresh orange (*Citrus* s*inensis*) juice was treated with crude enzymes 9.5:0.5 of juice to amylase. The control set-up contained distilled water in place of crude amylase [20]. Sodium benzoate 0.001 g was added to the experimental and control samples to inhibit microbial growth. The sample was incubated at 25 ± 3 ^o^C for 24 h, and clarification was monitored.

#### Starch liquefaction

Precisely 10 g of cassava starch was weighed into a 100-mL conical flask in duplicate. To the test experiment and control, 0.5 mL of crude amylase and 0.5 mL of water was added respectively, incubated at room temperature, and monitored for any physical changes for 8 h. Glucose released was monitored spectrophotometrically by dissolving aqueous glucose into the enzyme reagent with buffer solution (Tris buffer (pH 7.4) 92 mol/l, phenol 0.3 mol/l, glucose oxidase 1500 μ/l, peroxidase 1000 μ/l, 4-Aminophenozone 2.6 mol/l, glucose aqueous 100 mg/dl). The solution was mixed gently to ensure that all the reagent has gone into the solution before use.

### Statistical analysis

Data were presented as the mean of three experiments. SPSS software version 10.0 was used for the statistical analysis to determine the significant differences between the test variables using two-way ANOVA.

## Results

### Submerged fermentation of *Pleurotus tuber-regium* in cassava starch

*P. tuber-regium* was able to grow effectively on cassava starch, producing a viscous fermented liquid. *P. tuber-regium* is a versatile fungus that can utilize a variety of substrates. This is obvious in its successful utilization of soluble cassava starch.

### Partial purification of amylase

**Fig. 1:**
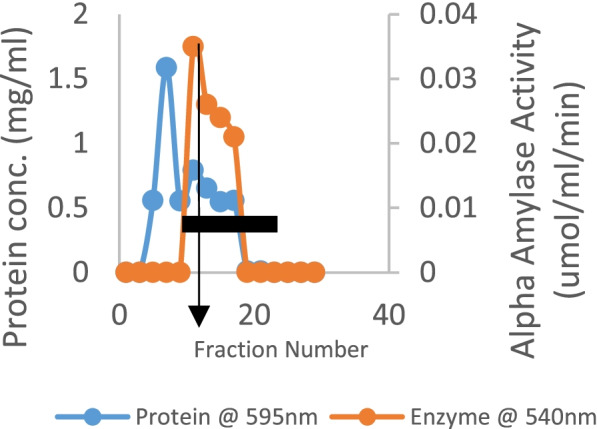
Purified amylase from *P. tuber-regium* on ion exchange chromatography

The crude amylase had a total activity of 610.29 U with a total protein content of 230.74 mg. The purification summary is presented in Table 1, signifying an increase in specific activity from 2.65 to 4.94 U/mg after ammonium sulfate precipitation. This step yielded a purification fold of 1.86 and a yield of 76.40 at 80% ammonium sulfate precipitation. Eluted fractions of the partially purified amylase on ion exchange chromatography are as shown in Fig. 1, with fractions 10–15 having enzyme activity with corresponding protein concentration.

**Table 1:** Summary of purification for alpha-amylase obtained from the submerged fermentation of *P. tuber-regium*

Fractions	Total Protein (mg)	Total Activity (U)	Specific Activity (U/mg)	Purification Fold	Yield (%)
Crude extract	230.74	610.29	2.65	1.00	100.00
Ammonium sulphate precipitation (80%)	95.17	470.12	4.94	1.86	76.40
CM sephadex c25 Ion-exchange chromatography	36.14	189.20	5.26	2.00	30.75

### Effect of pH on amylase activity

**Fig. 2:**
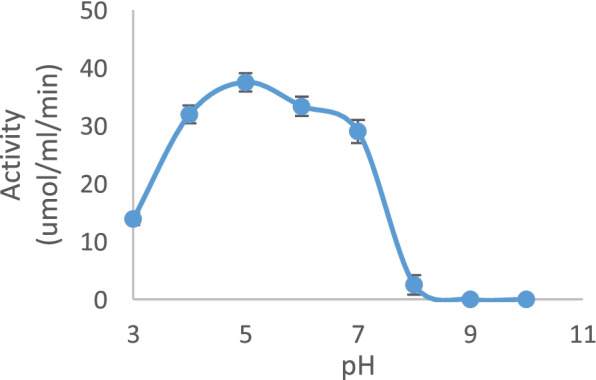
Effect of pH on amylase obtained from the submerged fermentation of *P. tuberregium*

The purified amylase activity from *P. tuber-regium* was determined over a pH range of 3 to 10, but activity was detected from 3 to 8. The optimum pH of the enzyme was observed to be at pH 5 (Fig. 2). This implies that *P. tuber-regium* amylase is active in the acidic pH range.

### Effect of temperature on amylase activity

**Fig. 3:**
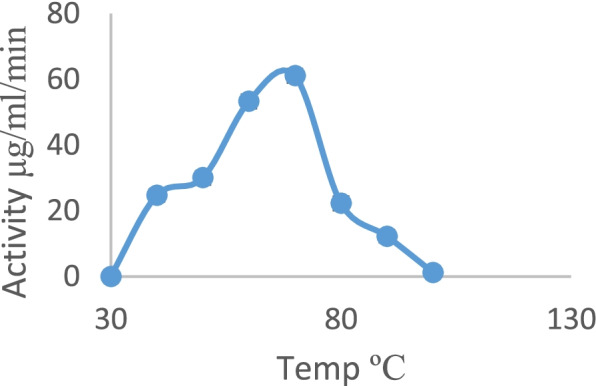
Effect of temperature on amylase obtained from the submerged fermentation of *P. tuberregium*

In this study, amylase activity was monitored at temperatures between 30 and 100 °C, with an optimum activity observed at 70 °C (Fig. 3). A decrease in enzyme activity was observed as the temperature increased from 70 to 100 °C.

### Heat stability of amylase from *P. tuber-regium*

Amylase from *P. tuber-regium* showed stability at 30 °C for 60 min with residual activity of 100%. The amylase retained 60% residual activity at 40 °C and 40% residual activity at 50 °C for about 30 min. *P. tuber-regium* lost most of its activity at 60 °C.

### Effect of metal salts on amylase activity

**Fig. 4:**
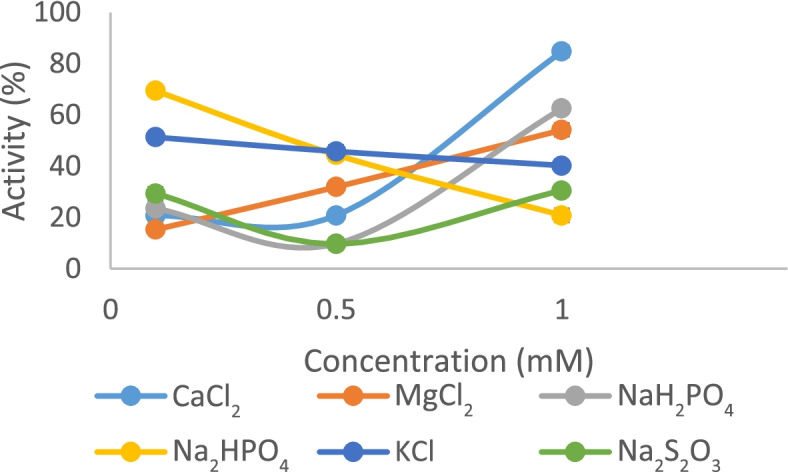
Effect of different concentrations of some compounds on amylase activity from *P. tuberregium*

The effect of some metal salts on amylase activity was determined. The results showed that some salts inhibited amylase activity while others enhanced enzyme activity. For instance, at concentrations of 0.1 and 1.0 mM, K^+^ slightly reduced enzyme activities from 50 to 40 μg/mL/min, respectively (Fig. 4). On the other hand, Ca^2+^ enhanced enzyme activities from 20 μg/mL/min at concentrations of 0.1 mM to 85 μg/mL/min at 1.0 mM (Fig. 4). In addition, Mg^2+^ enhanced enzyme activities from 15 to 55 μg/mL/min at concentrations of 0.1 and 1.0 mM respectively (Fig. 4).

### Substrate specificity

**Fig. 5:**
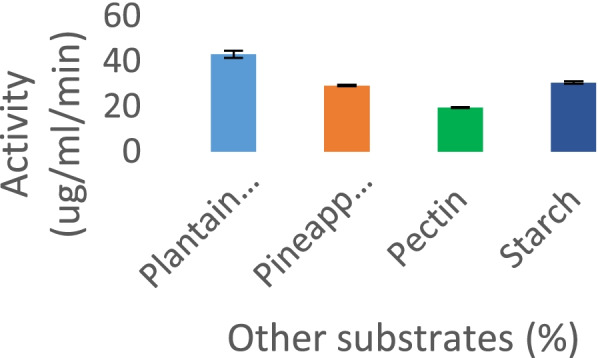
Effect of using other substrates on Amylase from *P. tuberregium*

The specificity of *P. tuber-regium* amylase for different substrates was studied. *P. tuber-regium* amylase showed optimal reaction with plantain peel (42 μg/mL/min). This specificity was followed by starch, pineapple peel, and pectin with 30, 29, and 27 μg/mL/min, respectively (Fig. 5).

### Biotechnological applications

#### Removal of sand and ink stain

Amylases are primarily used as carbohydrases for cleaning purposes. They improve cleaning efficiency by preventing the deposition of particles from soils rich in starch. The deinking process is used to eliminate unwanted contaminants from recycled paper materials. Recycling starts with the disintegration of used paper materials, followed by the separation of ink particles. The deinking process occurs in an alkaline medium, usually with chemicals. The use of enzymes instead of chemicals is faster, reduces environmental pollution, and is more efficient.

**Fig. 6:**
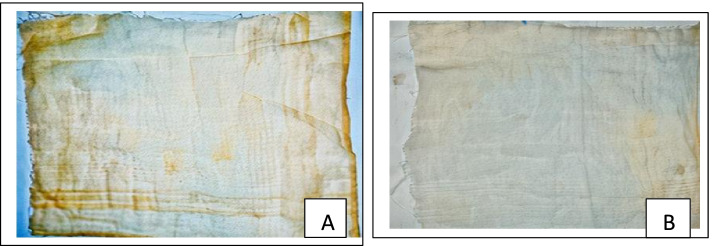
Wash performance of crude amylase of *P. tuberregium* on equally stained fabric (sand). **A** Water-washed fabric. **B** Cleaner fabric using crude amylase after 10 min

**Fig. 7:**
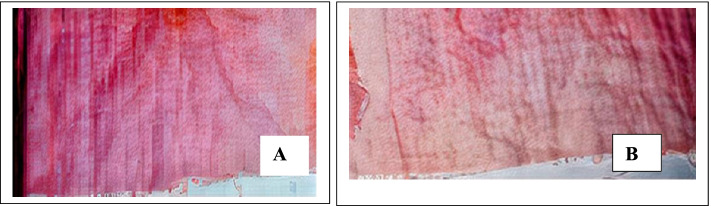
Wash performance of crude amylase on equally stained fabric (ink). **A** Water-washed fabric serving as the control. **B** Partially washed fabric with crude amylase after 10 min

In this study, at 10 min, a little stain was removed when the sand-stained fabric was washed with tap water, but when washed with crude amylase, the fabric got clearer than that of the control (Fig. 6). Also, the ink-stained fabric was clearer when washed with the crude amylase than when washed with water, which served as the control (Fig. 7).

#### Juice clarification and starch liquefaction

Fruit juices are usually cloudy in appearance due to complex polysaccharides such as starch and pectin [21]. This has necessitated enzymes including cellulase, pectic enzymes, amylases, and glucose oxidases. Enzymes are used to extract, clarify, and modify apple, pears, and citrus juices. Amylases are necessary biotechnology enzymes that digest or hydrolyses glycosidic bonds in starch and have been used commercially. Citrus fruits contain approximately 5% starch on a dry matter basis [22], affecting the turbidity of juices.

**Fig. 8:**
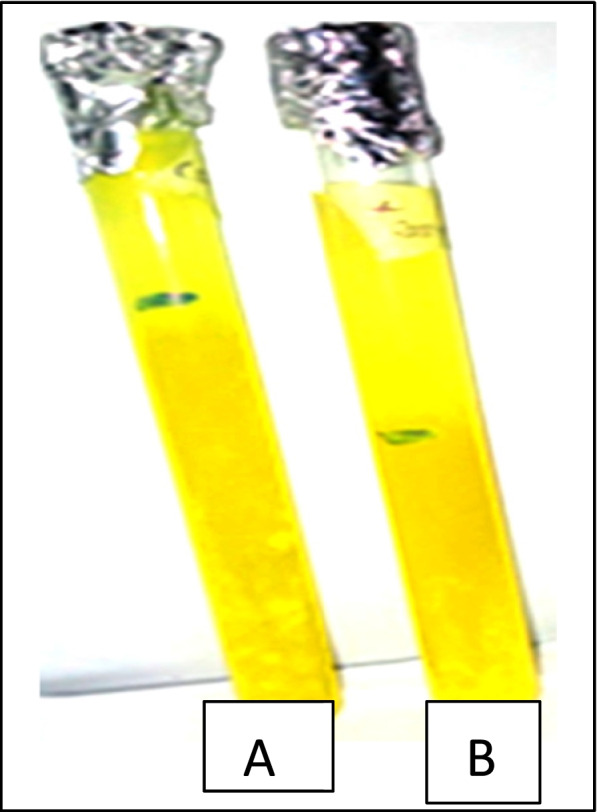
Amylase activity in juice clarification. **A** The control sample had little clarification (3 cm). **B** Better juice clarification with amylase (6 cm)

In this study, higher and better juice clarification was obtained for crude amylase than the control (containing water), which had a slight clarification, as some pulps were still visible in the upper layer of the juice (Fig. 8). The use of fresh orange for juice clarification with 0.001 g of sodium benzoate added to 9.5 mL of orange juice and 0.5 mL of crude amylase led to the production of clear juice, with the pulps settled at the tube base (Fig. 8). In this study, a two-fold clarification was obtained using crude amylase compared to water only.

**Fig. 9:**
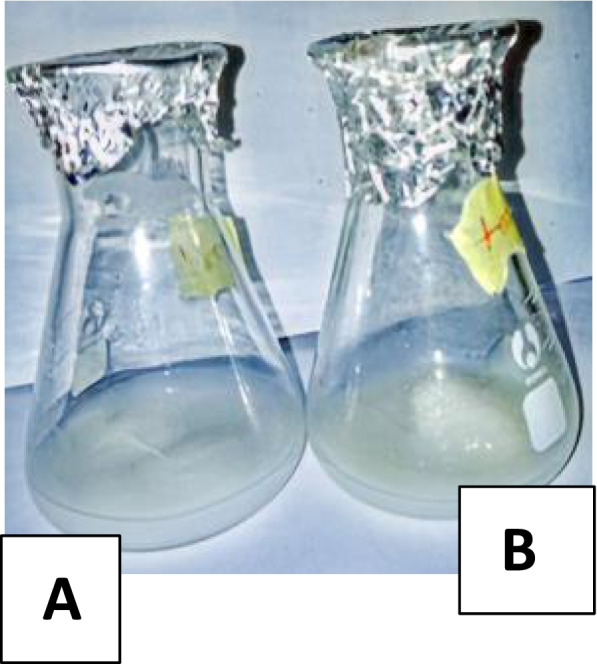
Liquefaction of cassava starch using crude amylase obtained from the submerged fermentation of *Pleurotus tuberregium*. **A** Glucose released from starch with the use of crude amylase yielded 99.25 mol/dm **B** Glucose released from control starch 67.17 mol/dm

As shown in Fig. 9, appreciable glucose quantity (99.25 mol/dm) was released from starch with crude amylase, while only 67.17 mol/dm was released in the control set-up.

## Discussion

The preference of the fungus understudy for starch has earlier been confirmed, as sclerotia formation is a gradual process that is stimulated by the breakdown of biomass and accumulation of breakdown products [14, 23].

In this study, amylase from *P. tuber-regium* was partially purified and characterized, and the biochemical properties determined using procedures that included ammonium sulfate precipitation and ion exchange chromatographic technique. The results showed a specific activity value of 5.26 U/mg with a percentage yield of 30.75 and a purification fold of 2.0. Different values have been reported for the purification yield of amylase from different sources. Krishnan and Chandra reported a percentage yield of 42% for purified *Bacillus licheniformis* CUMC305 amylase [24]. On the other hand, a percentage yield of 52% was reported for a thermostable amylase from a microbial source elsewhere [25]. A lower percentage yield of 6% was reported by Irshad et al*.* for *Ganoderma tsugae* [26]. Recently, a percentage yield of 18% was reported for *Trichoderma pseudokoningii* amylase [4]. This value is lower compared to the value obtained in this study. The differences in the percentage yields for the amylases could be related to differences in microbial strains and the purification procedures used.

Hydrogen ion concentration is one of the crucial factors that affect enzymatic activity. The highest activity of amylase in this study was obtained at a pH of 5.0. Similar results were reported for amylases from other sources. Khoo et al. reported a maximum activity at pH 4.2 for *A. niger* amylase. Further, they observed that the enzyme was stable in a pH range from 3.5 to 9.0 [27]. Similarly, a pH range of 5 to 10 was reported by Lim et al. working with a thermostable α-amylases from microbial sources [28]. A lower optimum pH range from 4.5 to 8.5 was reported for *Trichoderma pseudokoningii* amylase [4]. Other researchers have reported a pH range in the alkaline region of 7 to 9. Fungi amylases have been reported to have their optimum pH from 5.0 to 9.0. At the same time, bacterial showed a more comprehensive range of 5.0 to 10.5 [24]. For industrial application, pH stability is an essential factor. Therefore, amylase that would retain a significant portion of its activity at a particular pH would be a good candidate for such application. For example, *Aspergillus flavus* NSH9 was reported to retain 100% of its activity at pH 6.0, and 7.0 [29]. Most amylases are inactivated at the acidic pH range during starch processing. Therefore, amylases active in the acidic pH range are a good substitute [27]. Some other amylases have earlier been reported to have optimum activity at acidic pH [30–33] and neutral pH [34, 35].

The partially purified amylase from *P. tuber-regium* was observed to have the highest activity at 70 ^o^C. Different optimum temperatures have been reported for amylase enzymes from different sources. An optimum temperature of 70 °C was reported for *Bacillus* sp amylase [33]. A more thermophilic amylase was obtained from *A. penicillioides* with an optimum temperature at 80 ^o^C [36]. Others have reported lower optimum temperatures of 40 and 50 °C for amylases from *Lactobacillus plantarum* and *Tuber maculatum,* respectively [31, 37]. In this study, the amylase obtained from *P. tuber-regium* was stable at 30 ^o^C with 100% residual activity up to 60 min. *Bacillus* sp. amylase retained 75% of its activity at 75 °C for 45 min [38]. The amylase obtained from *Geobacillus thermoleovorans* retained 10% of its activity at 80 °C for 2 h without a substrate [39]. Krishnan and Chandra reported a highly stable amylase with stability up to 70% at 80 ^o^C for 90 min [24]. The amylase from this study could be a good source for industrial applications.

Ca^2+^ and Mg^2+^ ions were able to enhance the enzyme activity in this study, while K^+^ slightly inhibited its activity. A similar result was reported [4], where Ca^2+^ and Mg^2+^ notably enhanced amylase activity. These metal ions have been reported to inhibit amylase activity elsewhere [40, 37]. Some amylases are dependent on metal ions with Ca^2+^ in the active site. In such cases, Calcium ions improve the thermostability of amylases [41].

On substrate specificity, the amylase obtained in this study showed the highest activity with plantain peel, while the pectin as substrate showed the least activity. The enzyme was also able to utilize starch and plantain peel as substrates. These results are suggestive of the possible use of this amylase for industrial uses and with biodegradation potentials. Other studies have reported the specificity of amylases for different substrates. The substrate specificity profile is crucial because it characterizes and determines the kind of starch degraded most effectively and efficiently by amylase as a source of carbon [39]. Singh et al. reported that pomegranate peel and wheat bran were the best substrates for amylase production from *Aspergillus fumigatus* amylase [40]. Amylase from *A. thermarum* strain A4 with higher affinity for hydrolysis of amylose than other alpha glycosides was reported recently [42]. It was observed in another study that all substrate analogs used for *T. pseudokoningii* amylase had activity.

Amylase produced improved pulp brightness in deinking photocopied papers compared to cellulase [43]. In this study, a white fabric stained with sand was effectively unstained with crude amylase. A similar study reported that a white fabric stained with chocolate was cleaned [44], and tomato ketchup stain was also cleared [45]. In another study, alkaline amylase from *Monascus sanguineus* efficiently removed a mixture of spinach curry and chocolate [28, 46], while chocolate stain was removed in another study using amylase from *Bacillus subtilis* [47].

Consumers often desire clarification of fruit juices, which has led to the search for efficient and cost-effective clarification processes. Clarification of orange juice by up to two-fold was achieved using the crude amylase prepared in this study. Starch is one of the components of fruit juices that affects its turbidity. Orange juice contains about 76% carbohydrates of total dissolved solids, with significant starch content in its juice sac [48]. Enzymes from microbial sources are commonly exploited and preferred for juice clarification. Clarification of sweet orange [48], apple juice [49], and banana juice [50], with amylase has been reported.

## Conclusions

This study describes the isolation and characterization of amylase from the submerged fermentation of *P. tuberregium* using agro-waste as a nutrient source for the amylase production. The physicochemical properties of the *P. tuberregium* amylase showed that it could be a potential candidate for industrial applications.

## Data Availability

The datasets analyzed during the current study are available from the corresponding author on reasonable request.
